# Biological and clinical evidence for somatic mutations in *BRCA1* and *BRCA2* as predictive markers for olaparib response in high-grade serous ovarian cancers in the maintenance setting

**DOI:** 10.18632/oncotarget.17613

**Published:** 2017-05-04

**Authors:** Brian A. Dougherty, Zhongwu Lai, Darren R. Hodgson, Maria C.M. Orr, Matthew Hawryluk, James Sun, Roman Yelensky, Stuart K. Spencer, Jane D. Robertson, Tony W. Ho, Anitra Fielding, Jonathan A. Ledermann, J. Carl Barrett

**Affiliations:** ^1^ Innovative Medicines and Early Development, Oncology, AstraZeneca, Waltham, MA, USA; ^2^ Innovative Medicines and Early Development, Oncology, AstraZeneca, Cambridge, UK; ^3^ Personalized Healthcare and Biomarkers, AstraZeneca, Cambridge, UK; ^4^ Foundation Medicine, Inc., Cambridge, MA, USA; ^5^ Oncology Global Medicines Development, AstraZeneca, Cambridge, UK; ^6^ Oncology Global Medicines Development, AstraZeneca, Gaithersburg, MD, USA; ^7^ Oncology Global Medicines Development, AstraZeneca, Macclesfield, UK; ^8^ UCL Cancer Institute, London, UK

**Keywords:** BRCA, somatic, germline, olaparib, ovarian

## Abstract

To gain a better understanding of the role of somatic mutations in olaparib response, next-generation sequencing (NGS) of *BRCA1* and *BRCA2* was performed as part of a planned retrospective analysis of tumors from a randomized, double-blind, Phase II trial (Study 19; D0810C00019; NCT00753545) in 265 patients with platinum-sensitive high-grade serous ovarian cancer. *BRCA1/2* loss-of-function mutations were found in 55% (114/209) of tumors, were mutually exclusive, and demonstrated high concordance with Sanger-sequenced germline mutations in matched blood samples, confirming the accuracy (97%) of tumor *BRCA1/2* NGS testing. Additionally, NGS identified somatic mutations absent from germline testing in 10% (20/209) of the patients. Somatic mutations had >80% biallelic inactivation frequency and were predominantly clonal, suggesting that *BRCA1/2* loss occurs early in the development of these cancers. Clinical outcomes between placebo- and olaparib-treated patients with somatic *BRCA1/2* mutations were similar to those with germline *BRCA1/2* mutations, indicating that patients with somatic *BRCA1/2* mutations benefit from treatment with olaparib.

## INTRODUCTION

*BRCA1* and *BRCA2* mutational loss of function is a primary driver of breast and ovarian cancer and is also the basis of therapeutic treatment via a synthetic lethality mechanism of poly(ADP-ribose) polymerase (PARP) inhibition in conjunction with *BRCA1/2* or other homologous recombination genetic defects [1, 2]. The PARP inhibitor olaparib (Lynparza™, also known as AZD2281) is approved for ovarian cancer with germline *BRCA1/2* mutations in the US and EU, and for somatic *BRCA1/2* mutations in the EU only. *BRCA1* was originally identified based on linkage to genetic susceptibility in breast and ovarian cancers [3, 4]. In addition to germline mutations in *BRCA1*, a smaller proportion of somatic mutations in *BRCA1* were also found in primary ovarian and breast carcinomas [5, 6], and subsequent studies identified the *BRCA2* gene [7]. In high-grade serous ovarian cancer (HG-SOC), the most common subtype, *BRCA1/2* germline and somatic mutations are frequent (17-25%), with somatic mutations representing 18-30% of all *BRCA1/2* mutations [8, 9]. Assessment of the tumors of HG-SOC patients indicates that loss of the normal copy of *BRCA1/2* is observed in the majority of germline *BRCA1/2* mutations, consistent with this being an early event in the development of these cancers [6]. Recently, Pennington et al. [9] presented data suggesting that somatic mutations in homologous recombination genes, including *BRCA1/2*, result in a phenotype similar to tumors from patients with germline mutations in terms of differential overall survival (OS) and platinum sensitivity. However, the results were underpowered for statistical significance and patients with somatic *BRCA1/2* tumors were combined with those harboring mutations in other homologous recombination genes, making it difficult to determine the relative contribution of somatic *BRCA1/2* mutations.

Olaparib has activity against ovarian cancer in women with germline mutations in *BRCA1* or *BRCA2* [10], consistent with the synthetic lethality mechanism of PARP inhibitors [1, 2]. We have performed an investigation to: (i) use a sensitive next-generation sequencing (NGS) method to determine the mutational status of *BRCA1/2* in tumor tissues from patients in a randomized controlled trial of olaparib maintenance therapy in ovarian cancer; (ii) compare the results with known *BRCA1/2* mutational status from Sanger sequencing of blood samples; (iii) distinguish germline against somatic mutations and determine the genetic characteristics of somatic *BRCA1/2*-mutated tumors; and (iv) examine the responsiveness of somatically mutated tumors to PARP inhibition with olaparib relative to placebo.

## RESULTS

Tumor sections from 253 of the 265 participants in Study 19 (D0810C00019; NCT00753545) were submitted for NGS testing, and 209 (83%) passed all stages of quality control and resulted in successful sequencing results. As detailed in the Materials and methods, variants in *BRCA1* and *BRCA2* were identified, analyzed by the somatic germline zygosity (SGZ) algorithm [11] and classified according to American College of Medical Genetics and Genomics (ACMG) standards [12]. When combined with germline testing, results demonstrated an overall 55% (114/209) *BRCA1/2* mutation rate (Table 1 and Supplemental Table 1). The concordance between the germline-mutation-positive patients identified by Sanger sequencing and the NGS-based tumor test was 97% (71/74). The three discordant patients, classified as non-mutant by the tumor NGS assay but positive by germline blood testing, were confirmed as mutant by visual inspection of aligned NGS data (Supplemental Figure 1). These mutations (one *BRCA1* exon deletion, two *BRCA1* exon duplications) were below the Foundation Medicine T5 NGS assay limit of detection for single exon alterations.

**Table 1:** Concordance of tumor NGS *BRCA1/2* sequencing test status with blood germline Sanger testing for the 209 Study 19 patients with tumor testing results

		Tumor (somatic) *BRCA1/2* mutation status					
		Mutant (n=111)			VUS (n=12)	Non-mutant (n=86)	Total
		Germline	Somatic	Unknown			
**Blood (germline) B*RCA1/2* mutation status**	**Mutant**	71	–	–	–	3	**74**
	**VUS**	–	–	–	4	–	**4**
	**Non-mutant**	–	14	–	4	66	**84**
	**Not tested**	19	6	1	4	17	**47**
	**Total**	**90**	**20**	**1**	**12**	**86**	**209**

Tumor testing (Foundation Medicine T5 panel NGS assay) resulted in calls of mutant, VUS, or non-mutant and was compared with germline testing (Myriad Integrated BRACAnalysis^®^ or CRF [case report form]) for the same categories, as well as not tested. A total of 114 mutated patient tumors were identified, with 90 germline, 20 somatic, one of unknown germline/somatic origin, and three germline mutant but originally called non-mutant by the tumor assay. No mutations identified by germline testing were predicted by the tumor test as somatic. Note that there is one patient with somatic VUS tumor status harboring two somatic VUS – this patient was not tested by blood germline testing.

**Figure 1:**
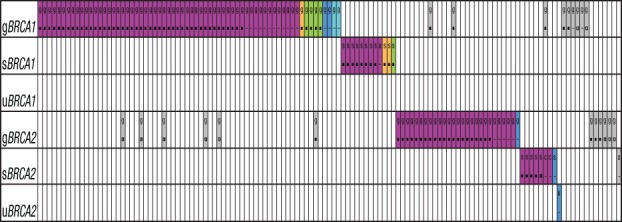
Mutual exclusivity of *BRCA1* and *BRCA2* mutations The subset of samples with *BRCA1/2* loss-of-function mutations (n=114) and with only VUS (n=13) are plotted by patient (column) and by gene and origin of gene mutation (row) as germline (g*BRCA*), somatic (s*BRCA*), or of unknown origin (u*BRCA*). Mutations are color coded by type (purple for frameshift or nonsense truncating, orange for splice site, green for clinically important missense, dark blue for homozygous deletions, light blue for insertions/rearrangements, gray for VUS) and zygosity (■ for homozygous, ◨ for heterozygous, □ for not in tumor, - for unknown, c for subclonal somatic). Note that while *BRCA1/2* loss-of-function mutations (purple, orange, green, dark blue, light blue) are mutually exclusive, VUS (gray) can occur concomitantly with *BRCA1/2* loss-of-function mutations. Not all VUS are represented because of co-occurrence with loss-of-function mutations or VUS in the same gene; a complete listing is found in Supplemental Table 1.

Of the 114 patient tumors classified as mutant by the tumor test, SGZ analysis combined with comparison of blood and tumor assay results predicted 93 germline mutations, 20 somatic mutations, and one mutation of unclear germline/somatic origin that was not tested by the germline assay (Table 1 and Supplemental Table 1). All germline and somatic *BRCA1* and *BRCA2* loss-of-function mutations were mutually exclusive (Figure 1). Categorization of the 114 mutant tumors (78 *BRCA1* and 36 *BRCA2*) by mutation type determined that 94% of mutations identified were short variants, with the predominant mutation type being frameshift mutations (Supplemental Figure 2 and Supplemental Table 1). SGZ predictions were consistent with blood testing, as 71 predicted germline mutations were confirmed as germline mutant; for the remainder, six were designated non-mutant because of incomplete testing, 13 were not tested, and three were not called mutant in the original analysis (see above). For the 20 patients predicted to have somatic mutations in their tumors by SGZ, 14 were found non-mutant by germline testing and six were not tested (Supplemental Table 1 and Supplemental Figure 3).

The germline mutations demonstrated a 100% biallelic inactivation rate in the tumors since the 71 mutations for which zygosity could be called were all homozygous (the zygosity of 17 short variant germline mutations could not be determined; Supplemental Figure 3 and Supplemental Table 1). The subset of somatic mutations had an 83% biallelic inactivation rate (of 20 somatic mutations, 18 were called by SGZ and 15 demonstrated biallelic inactivation), with intact copies of *BRCA2* in a tumor with a heterozygous mutation (patient AZ-19-4449) and two tumors which harbored subclonal somatic mutations (Supplemental Table 1). By contrast, the *BRCA1/2* variants of unknown significance (VUS) demonstrated a 43% (13/30 callable) biallelic inactivation rate, with 13 homozygous variants versus five heterozygous and 12 not in tumor; the zygosity of four VUS could not be determined, including one patient (AZ-19-4313) with two somatic VUS in the same tumor (Supplemental Table 1). Only eight patients lacking *BRCA1/2* loss-of-function mutations harbored a VUS that was homozygous or not callable by SGZ, suggesting a limited number of VUS that may possibly impact *BRCA1/2* function in this cohort (Figure 1 and Supplemental Table 1). SGZ analysis also confirmed a high rate of *BRCA1/2* mutation clonality for mutant tumors since all loss-of-function mutations were predicted clonal, with the exception of the two patients with predicted subclonal somatic mutations. Furthermore, somatic *BRCA1/2* mutant allele frequencies relative to those of co-occurring *TP53* mutations also demonstrated a correlation consistent with few subclonal *BRCA1/2* mutation events among Study 19 patients (Supplemental Figure 4).

**Figure 2:**
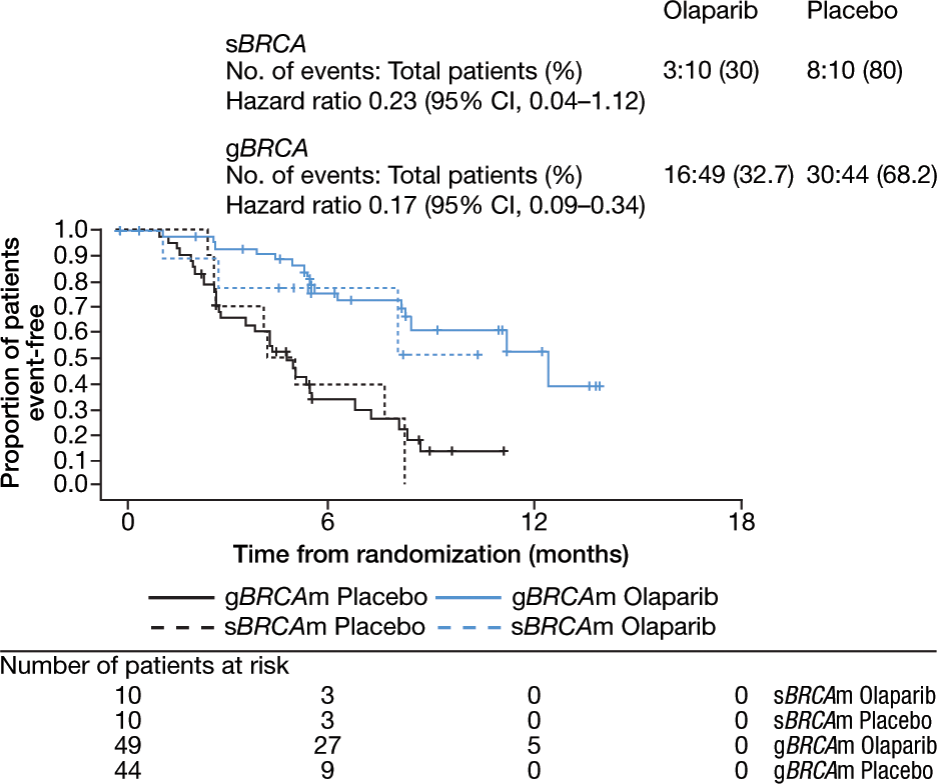
Progression-free survival of *BRCA1/2* somatic- *versus* germline-mutated patients Time for somatic-mutated (s*BRCA*m) and germline-mutated (g*BRCA*m) patients treated with olaparib (blue line) or placebo (black line) is plotted against the proportion of patients event-free.

The impact of combined germline and somatic *BRCA1/2* mutational status on clinical outcomes was previously reported for Study 19 patients [10] and showed a clinically large and statistically significant treatment effect for olaparib versus placebo (progression-free survival [PFS] hazard ratio [HR] 0.18, 95% confidence interval [CI] 0.10-0.31, *P*<0.0001). We performed an exploratory clinical outcomes analysis on the patients identified in this study as having somatic or germline *BRCA1/2* mutations. The large magnitude of the treatment effect in patients with somatic *BRCA1/2* mutations (PFS HR 0.23, 95% CI 0.04-1.12) was consistent with that observed in patients identified based on tumor analysis as having germline *BRCA1/2* mutations (PFS HR 0.17, 95% CI 0.09-0.34; Figure 2 and Table 2), as well as that previously reported in all patients with *BRCA1/2* mutations [10]. Additionally, the exploratory analyses of OS, time to first subsequent therapy or death (TFST), time to discontinuation or death (TDT), and time to second subsequent therapy or death (TSST) all demonstrated trends favoring olaparib versus placebo treatment for patients with platinum-sensitive HG-SOC with somatic *BRCA1/2* mutations (Table 2), consistent with observations in patients with germline *BRCA1/2* mutations based on tumor analysis and in all patients with *BRCA1/2* mutations, as previously reported [10].

**Table 2:** Progression-free survival, overall survival, time to discontinuation of treatment, and time to first and second subsequent therapy for somatic- and germline-*BRCA1/2*-mutated patients with platinum-sensitive high-grade serous ovarian cancer receiving olaparib 400 mg twice daily or placebo in Study 19 (data cut-off November 26, 2012)

Cohort	Endpoint	Treatment	N	Number of events (%)	HR	95% CI
Somatic *BRCA*	PFS^a^	Olaparib	10	3 (30)	0.23	0.04, 1.12
		Placebo	10	8 (80)		
	OS	Olaparib	10	3 (30)	0.15	0.02, 0.88
		Placebo	10	7 (70)		
	TDT	Olaparib	10	7 (70)	0.23	0.05, 0.93
		Placebo	10	10 (100)		
	TFST	Olaparib	10	6 (60)	0.48	0.12, 1.91
		Placebo	10	8 (80)		
	TSST	Olaparib	10	5 (50)	0.39	0.08, 1.63
		Placebo	10	8 (80)		
Germline *BRCA*	PFS^a^	Olaparib	49	16 (32.7)	0.17	0.09, 0.34
		Placebo	44	30 (68.2)		
	OS	Olaparib	49	24 (49.0)	0.62	0.34, 1.12
		Placebo	44	22 (50.0)		
	TDT	Olaparib	49	39 (79.6)	0.37	0.23, 0.59
		Placebo	44	41 (93.2)		
	TFST	Olaparib	49	30 (61.2)	0.33	0.20, 0.54
		Placebo	44	38 (86.4)		
	TSST	Olaparib	49	27 (55.1)	0.39	0.23, 0.66
		Placebo	44	33 (75.0)		

^a^Data cut-off June 30, 2010. N, number of patients with a mutation

## DISCUSSION

The standard accepted methodology for determining *BRCA1* and *BRCA2* mutational status has been Sanger sequencing of germline genomic DNA following amplification by polymerase chain reaction of exons and nearby intronic DNA. It is challenging to perform cost effectively complete and accurate sequencing of these large tumor suppressor genes, and Sanger sequencing approaches are not fit for detecting the low allele frequencies encountered when sequencing small amounts of low-quality DNA from formalin-fixed tissue with variable tumor cellularity. In this study of platinum-sensitive, relapsed HG-SOC, NGS was performed on hybridization-capture-enriched, formalin-fixed paraffin-embedded (FFPE)-derived tumor genomic DNA and accurately identified *BRCA1/2* mutations. Of the 93 known or predicted germline mutations, only three were not identified using the tumor NGS assay. This is because of the difficulty in algorithmically detecting single exon deletion and insertion/duplication events, and it is likely that this class of mutation is currently being under-reported by most NGS-based *BRCA1/2* assays, in particular, tumor assays using clinical FFPE samples of low tumor cellularity. Additional studies are needed to confirm the 97% concordance seen in this study. Optimization of NGS assays may help further increase the accurate identification of structural rearrangements through explicit capture of common breakpoint locations or other approaches.

Attention to detail is required not only when comparing two sequencing assays, but also in the categorization of mutations, where occasional discrepancies may occur. For instance, the *BRCA1* R1699Q missense variant is considered a VUS in the Breast Cancer Information Core (BIC) database but a pathogenic mutation in Online Mendelian Inheritance in Man and the published literature [13, 14], and pathogenic and likely pathogenic in ClinVar (entries RCV000031217, RCV000048790, RCV000131564, RCV000195350). Our results are similar to those of another study comparing a germline NGS assay with Myriad Sanger sequencing, whereby 57 of 59 *BRCA1/2* mutations were determined to be concordant, with the two discrepancies being a VUS classification issue and a large DNA insertion event [15].

The present study analyzes the Study 19 *BRCA1/2* mutational data from a tumor-based perspective, focusing on the 209 patients with tumors successfully sequenced by NGS. NGS-based testing of tumor tissue enables the identification of somatic mutations that are not found by blood-based testing methods and that are often not detected at low allele frequencies by Sanger sequencing of tumor samples. The tumor assay used here identified 10% (20/209) of patients with somatically mutated tumors that would not be detected by germline testing and enables *BRCA1/2* testing of clinical FFPE specimens with a sensitivity, cost, and sample input that cannot be achieved by Sanger sequencing techniques. Furthermore, the ability of the assay to perform mutational analysis of hundreds of cancer genes at once provides insight into additional oncogenic drivers, mutational co-occurrence patterns, tumor heterogeneity, and the potential to identify homologous recombination and DNA repair genes beyond *BRCA1* and *BRCA2* that may be involved in response to olaparib and other PARP inhibitors.

SGZ analysis of tumor NGS data indicated high rates of biallelic inactivation for *BRCA1/2* mutations, suggesting that loss of *BRCA1/2* function is an early event in the progression of ovarian cancer. Biallelic inactivation was much more frequent among germline (100%) and somatic (83%) *BRCA1/2* loss-of-function mutations, as opposed to VUS (43%). Most VUS (20/34) co-occurred with biallelic *BRCA1/2* loss-of-function mutations and thus are likely not responsible for loss of gene function. Given that only eight *BRCA1/2* non-mutant tumors harbored homozygous VUS or VUS of unknown zygosity, this represents a small set of candidate VUS that may possibly impact *BRCA1/2* gene function (Supplemental Table 1). Thus, while VUS are often the topic of much research into predicting functional impact, the combined computational analysis of zygosity, germline/somatic origin, and co-occurrence with loss-of-function *BRCA1/2* mutations presented here may help to deprioritize many of the VUS that are likely rare germline single nucleotide polymorphisms (SNPs) or somatic passengers that do not impact *BRCA1/2* function.

The 55% rate of *BRCA1/2* mutations in the Study 19 tumor-sequenced subpopulation was higher than the 20-26% rate seen in other HG-SOC tumor-sequencing studies (Supplemental Table 2). The high *BRCA1/2* mutation frequency is not completely explained by platinum sensitivity and may have been influenced by local knowledge of germline-mutated *BRCA1/2* status for 49 patients. However, the 18% (20/114) of mutated tumors with somatic *BRCA1/2* mutations for Study 19 is the same as for the HG-SOC subset of the Pennington et al*.* cohort (Supplemental Table 2). The 30% somatic mutation rate of The Cancer Genome Atlas (TCGA) ovarian cohort is higher than in both studies, but there are potential selection biases in the TCGA cohort (discussed by Pennington et al. [9]). The rate of somatic *BRCA1/2* mutations in other cancers may differ from those for ovarian cancer. A recent study in an unselected breast cancer cohort reported somatic-only *BRCA1/2* mutations to be present in 3% of patients, with approximately one-third (9/29) of *BRCA* mutations to be of somatic origin [16]. Similarly, the 482-subject TCGA breast cohort reported an 8% *BRCA1/2* mutation rate with 34% somatic mutations, with enrichment to 20% *BRCA1/2* mutations within the basal subtype [17]; however, both of these studies have potential selection biases similar to the TCGA ovarian cohort which may result in variable estimates for both germline and somatic mutation rates. In a study of aggressive, treatment-naïve advanced prostate cancer assessing *BRCA1/2* mutations by whole genome sequencing, a 0.6% *BRCA1* and 12% *BRCA2* mutation rate was observed, all with biallelic inactivation, and half of which were somatic mutations [18]. The authors noted that the contribution of difficult-to-detect variant types, paired with the fact that tumor sequencing studies often do not systematically assess germline alleles, might underlie the low reported frequency of *BRCA2* mutations in earlier prostate cancer sequencing studies.

In this analysis of Study 19, we have distinguished somatic from germline *BRCA1/2* mutations by tumor sequencing and analysis in order to test the hypothesis that somatic *BRCA1/2* mutations should have similar functional effects to germline mutations. This hypothesis was investigated using a sensitive and reliable method to determine the mutational status of *BRCA1/2* in tumor tissues from a pivotal study with olaparib, providing the opportunity to determine the clinical activity of olaparib on somatically mutated *BRCA1/2* while also benchmarking the accuracy of NGS compared with the standard of Sanger sequencing methodology. Using tumor SGZ classification and comparison with germline assay results, we identified 20 somatic *BRCA1/2* mutation cases, a slightly larger number than the 18 candidate somatic-*BRCA1/2*-mutated patients in our previous report [10]. Among the 20 somatic-*BRCA1/2*-mutated tumor samples, there was one heterozygous *BRCA2* mutation that lacked clear biallelic inactivation at the time of archival tumor collection prior to the Study 19 trial and two *BRCA2* mutations predicted as subclonal somatic by the SGZ algorithm. The predicted subclonal somatic *BRCA2* mutations are worthy of further examination to determine if these mutations became clonal drivers by the time of treatment with platinum or olaparib. It is notable that the two patients with subclonal *BRCA2* mutations in their archival biopsies (AZ-19-4349, AZ-19-4367) were on olaparib treatment for over 2 years, suggestive of treatment benefit (unpublished observations).

Although not statistically robust owing to the small numbers, analysis of clinical outcomes for somatic-*BRCA1/2*-mutated patients demonstrated trends favoring olaparib over placebo. The current data support the hypothesis that the majority of somatic-*BRCA1/2*-mutated cases should have a similar biological phenotype to germline-*BRCA1/2*-mutated tumors. The clonality and mutated allele fractions relative to *TP53* suggest the presence of the mutation in all tumor cells, and the mutual exclusivity with germline mutations suggests phenotypic redundancy. Although patient numbers were small, perhaps most importantly, the clinical outcomes for somatic-*BRCA1/2*-mutated patients in this randomized controlled trial of olaparib are consistent with those for the germline-mutated patients. In a single-arm study, Swisher et al. [19] and Konecny et al. [20] recently reported a similar response rate in patients with germline (n=32) and somatic (n=19) mutant *BRCA1/2* HG-SOC (who had received 3-4 lines of prior treatment) treated with rucaparib. Mirza et al. [21] reported a similar HR for PFS in patients with somatic- (n=47) and germline-treated germline *BRCA1/2* mutations. Our data further support the clinical benefit of a PARP inhibitor in treated somatically mutated *BRCA1/2* patients (n=20) being similar to that seen in germline-mutated patients in terms of both PFS and longer-term measures of benefit, such as OS. Given that somatic-*BRCA1/2*-mutated individuals may represent 20-30% of HG-SOC patients with *BRCA1/2* mutations, full characterization of the effects of PARP inhibitors in larger patient populations is an important clinical question deserving further research.

## MATERIALS AND METHODS

### Tumor DNA sequencing

Tumor samples were available from 253 of the 265 patients randomized in the olaparib Study D0810C00019 (Study 19; ClinicalTrials.gov NCT00753545 [22]). The study was performed in accordance with the Declaration of Helsinki, Good Clinical Practice, and the AstraZeneca policy on bioethics. The Foundation Medicine FoundationOne™ (T5 panel) NGS assay [23] was used to perform a pre-specified retrospective genetic analysis of tumor samples. Briefly, tumor samples from each patient were accessioned and underwent pathology review, and DNA was purified from eight 5-μm FFPE sections containing at least 20% tumor, without any macrodissection or tumor enrichment. Libraries were prepared for Illumina sequencing with 50-200 ng of genomic DNA, and hybridization-capture baits were used to enrich for coding regions of 287 cancer-related genes and additional genomic locations. Bioinformatic analysis was performed to align sequence reads and call short variants, copy number gains, homozygous deletions, and select translocations. Of the 253 tumor samples, there were 44 non-reported samples that failed at the following quality control checkpoints: 8% (20/253) tissue insufficient for analysis; 2% (5/253) at DNA purification; 3% (8/253) at library construction; and 4% (11/253) at sequence coverage and quality.

### Germline DNA sequencing

Germline *BRCA1/2* mutation status was determined by a local sequencing test in the CRF and/or retrospectively using the Integrated BRACAnalysis assay (Myriad Genetics Laboratories, Inc., Salt Lake City, UT, USA; https://www.myriad.com/lib/technical-specifications/BRACAnalysis-Technical-Specifications.pdf) with DNA extracted from blood samples collected before randomization. The *BRCA1/2* genes were sequenced and examined for mutations and rearrangements (deletions and duplications) in the coding regions and 10-20 base pairs of flanking intronic sequence. Patients were defined as germline *BRCA1/2* mutated if they harbored a deleterious, or suspected deleterious, *BRCA1/2* mutation in their germline DNA in accordance with the standards outlined by the ACMG.

### Bioinformatic analysis of tumor sequencing

After filtering for common polymorphisms, 142 *BRCA1/2* short coding variants were confirmed by visual inspection of .bam alignment files and classified using ACMG standards [12] as mutant (deleterious or suspected deleterious), VUS, or non-mutant (benign or suspected polymorphism, or no variant detected within the gene, often referred to as wild type). All loss-of-function variants (frameshift, nonsense, essential splice site), as well as homozygous deletions of exons and insertions/duplications of exons, were considered mutant, whereas all missense variants were initially classified as VUS and then, following comparison with the BIC database [24], re-classified as mutant (five samples) or non-mutant (one sample, benign SNP *BRCA2* S326R) if clinical significance was known in the BIC database (variants listed as of December 5, 2012). One variant (R1699Q) classified as VUS by BIC was designated as mutated given supportive literature data [13, 14] and ClinVar entries (RCV000031217, RCV000048790, RCV000131564, RCV000195350).

### Somatic germline zygosity analysis

The SGZ classifier [11] was developed in order to help predict the origin of the short variant mutation (germline, somatic, or unknown) and the zygosity (homozygous, heterozygous, not in tumor, subclonal somatic, or unknown) using only information derived from sequencing and analysis of a tumor sample. The presence of a homozygous *BRCA1/2* mutation was considered a loss-of-heterozygosity event resulting in biallelic inactivation.

The predicted germline and somatic status from tumor DNA sequencing was compared with *BRCA1/2* germline testing, reported in the CRF and/or performed by Myriad Genetics BRACAnalysis of matched 10-mL blood samples. Of the original 265 patients in Study 19, germline *BRCA1/2* status was determined locally via CRF for 98 consented patients (six were excluded because of incomplete or inconclusive results) and centrally via Myriad for 174 patients (14 samples were excluded because of inadequate DNA yields or incomplete sequence data). We previously reported 18 patients with candidate somatic *BRCA1/2* mutations in their tumors based on comparing tumor and blood data, either from central testing at Myriad (n=10) or locally reported via CRF (n=8), and a further 22 patients with mutations in their tumor but for whom no blood testing data were available [10]. The new analysis reported here includes six candidate somatic-*BRCA1/2*-mutated tumors from the group of 22 patients for whom no blood testing data were available and 14 of the 18 candidate somatic-*BRCA1/2*-mutated patients previously reported. Hence, four patients from the previously reported group of 18 somatic-*BRCA1/2*-mutated patients are not included, three owing to likely incomplete CRF-reported local blood-based *BRCA1/2* testing and the fourth because of discordant variant results revealing that the blood and tumor samples were from different individuals.

### Statistical analysis of clinical outcomes

A total of 20 somatic-*BRCA1/2*-mutated patients (10 on olaparib and 10 placebo treated) were identified by SGZ analysis coupled with analysis of blood versus tumor sample testing results, with 14 having supportive non-mutant germline results and six not tested. The somatically mutated patients were analyzed for PFS, OS, TFST, TDT, and TSST. HRs and corresponding CIs were calculated from a Cox proportional hazards model using the same methods as reported for the primary analysis [10].

## SUPPLEMENTARY MATERIALS FIGURES AND TABLES





## Abbreviations

ACMG: American College of Medical Genetics and Genomics
BIC: Breast Cancer Information Core
CI: confidence interval
CRF: case report form
FFPE: formalin fixed paraffin embedded
HG-SOC: high-grade serous ovarian cancer
HR: hazard ratio
NGS: next-generation sequencing
OS: overall survival
PARP: poly(ADP-ribose) polymerase
PFS: progression-free survival
SGZ: somatic germline zygosity
SNP: single nucleotide polymorphism
TCGA: The Cancer Genome Atlas
TDT: time to discontinuation
TFST: time to first subsequent therapy or death
TSST: time to second subsequent therapy or death
VUS: variant of unknown significance.
