# Supplementary material for: Biological and clinical evidence for somatic mutations in BRCA1 and BRCA2 as predictive markers for olaparib response in high-grade serous ovarian cancers in the maintenance setting
Source: Oncotarget. 2017 May 4;8(27):43653–61. doi: 10.18632/oncotarget.17613 (PMC5546431; doi:10.18632/oncotarget.17613)
Supplement: Supplementary file 1 [file oncotarget-08-43653-s001.pdf]

Biological and clinical evidence for somatic mutations in *BRCA1* and *BRCA2* as predictive markers for olaparib response in high-grade serous ovarian cancers in the maintenance setting

Supplementary Material

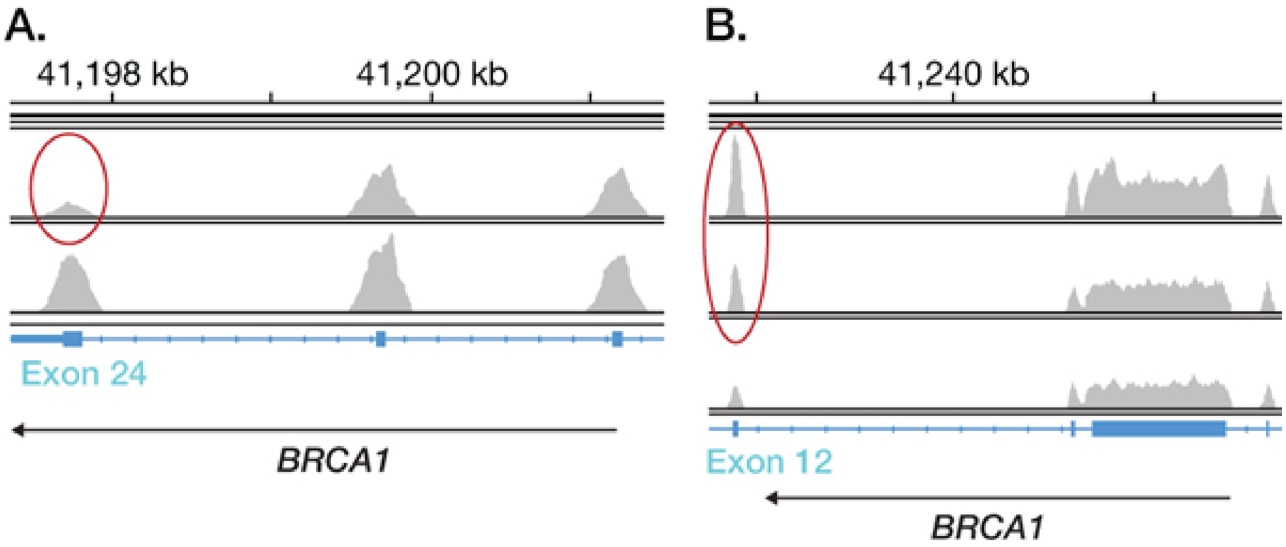

Supplemental Figure 1: Sequencing coverage plots of three samples with *BRCA1/2*-mutated exons found by comparison with blood testing results. (A) Deletion of exon 24 in AZ-19-4224 tumor relative to non-mutant AZ-19-4223. (B) Insertion/duplication of 6 kb into exon 13 in AZ-19-4393 and AZ-19-4463 tumors relative to non-mutant AZ-19-4242. Sequencing coverage scaled to 1500 reads in IGV genome viewer.

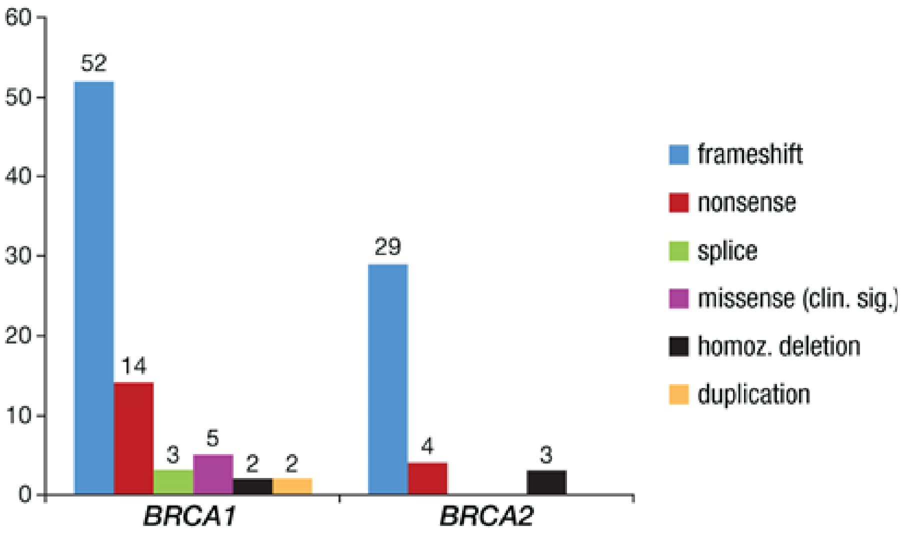

Supplemental Figure 2: ***BRCA1*** **and** ***BRCA2*** **mutations categorized by type.** Loss-of-function mutations included short variants (frameshift, blue; nonsense, red; splice site, green; clinically relevant missense, purple) as well as homozygous deletions (black) and duplications (yellow)

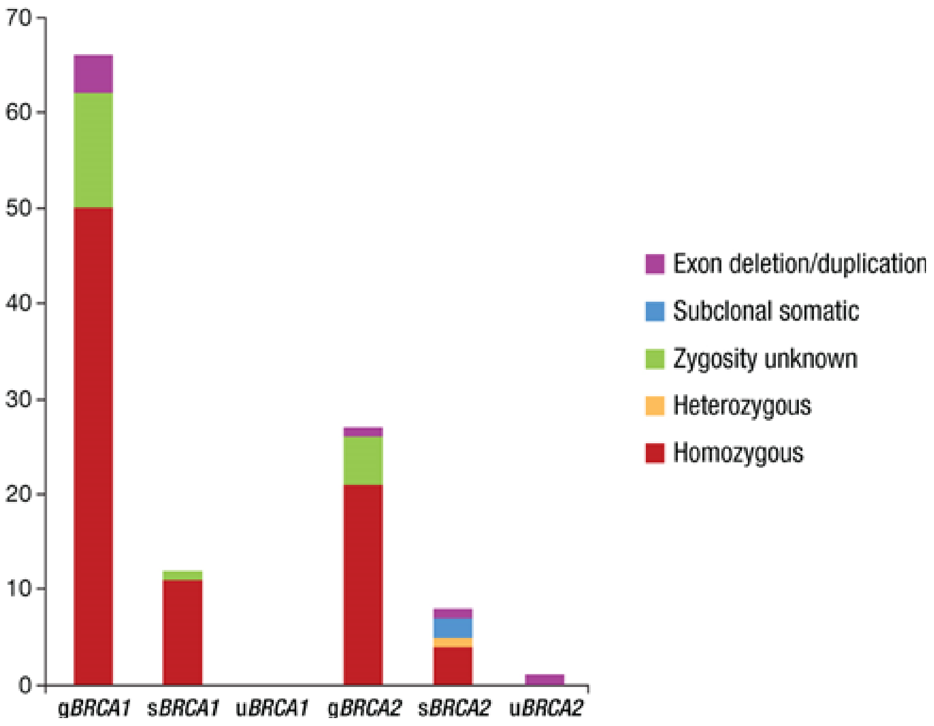

Supplemental Figure 3: Zygosity of ***BRCA1/2*** **mutations by germline/somatic origin.** Mutation zygosity of short variants (homozygous, red; heterozygous, yellow; subclonal somatic, blue; zygosity unknown, green) as well as exon-level homozygous deletions and duplications (purple) were compared with origin of mutations (germline, somatic, or unknown) and gene (*BRCA1* or *BRCA2*)

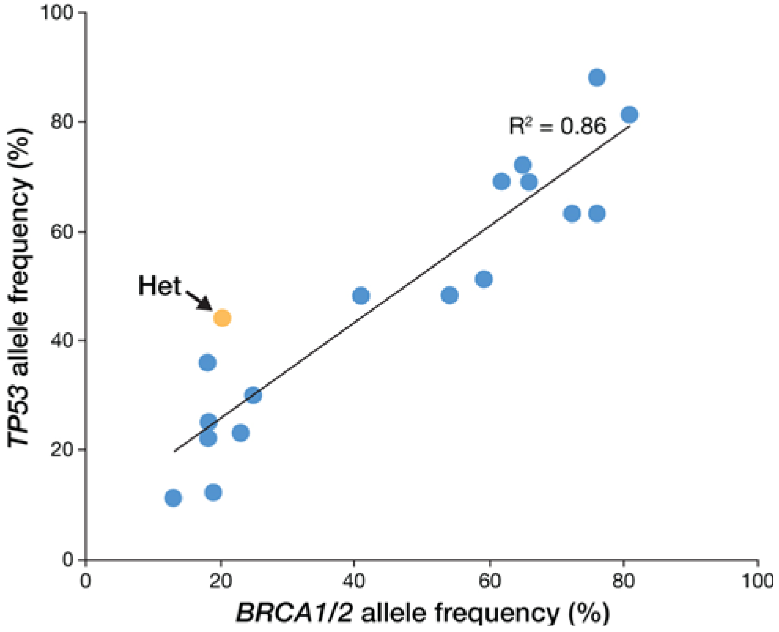

Supplemental Figure 4:Correlation of allele frequencies for somatic *BRCA1/2* mutations and *TP53* mutations. Data from two of the 20 somatic-*BRCA1/2*-mutated tumors are not plotted, one with a *BRCA2* deletion of unknown allele frequency and one lacking a *TP53* mutation

For Supplementary Tables 1, 2 see in supplementary Files.
