# Supplementary material for: Biological and clinical evidence for somatic mutations in BRCA1 and BRCA2 as predictive markers for olaparib response in high-grade serous ovarian cancers in the maintenance setting
Source: Oncotarget. 2017 May 4;8(27):43653–61. doi: 10.18632/oncotarget.17613 (PMC5546431; doi:10.18632/oncotarget.17613)
Supplement: Supplementary file 2 [file oncotarget-08-43653-s002.docx]

**Biological and clinical evidence for somatic mutations in *BRCA1* and *BRCA2* as predictive markers for olaparib response in high-grade serous ovarian cancers in the maintenance setting**

Brian A. Dougherty, Zhongwu Lai, Darren R. Hodgson, Maria C. M. Orr,
Matthew Hawryluk, James Sun, Roman Yelensky, Stuart K. Spencer, Jane D. Robertson, Tony W. Ho, Anitra Fielding, Jonathan A. Ledermann and J. Carl Barrett

**Supplemental data**

**Supplemental Table 1.** Mutation status for 209 Study 19 patients with tumor assay results. Each patient tumor has a tumor mutation classification (*BRCA1/2* loss-of-function mutation of germline, somatic, or unknown origin; VUS; or non-mutant), mutation type, amino acid change, predicted germline or somatic status, and predicted zygosity of mutations, compared with germline assay test type (CRF, Myriad, both, or not tested) and assay result (mutant, non-mutant, VUS, not tested)

| **Patient** | **Tumor *BRCA1* test** | | | | **Tumor *BRCA2* test** | | | | **Germline *BRCA1/2* test** | |
| --- | --- | --- | --- | --- | --- | --- | --- | --- | --- | --- |
| **Classification** | **Mutation type** | **Amino acid change** | **Germline/ somatic** | **Zygosity** | **Mutation type** | **Amino acid change** | **Germline/ somatic** | **Zygosity** | **Germline assay test** | **Germline assay classification** |
| g*BRCA1*m | Frameshift | Q1756fs*74 | Germline | Homozygous |  |  |  |  | CRF | Mutant |
| g*BRCA1*m | Frameshift | E23fs*17 | Germline | Homozygous |  |  |  |  | CRF | Mutant |
| g*BRCA1*m | Frameshift | E23fs*17 | Germline | Homozygous |  |  |  |  | Both | Mutant |
| g*BRCA1*m | Frameshift; VUS | V340fs*1; E575K | Germline; germline | Homozygous; homozygous |  |  |  |  | Both | Mutant |
| g*BRCA1*m | Frameshift | Q1756fs*74 | Germline | Homozygous |  |  |  |  | CRF | Mutant |
| g*BRCA1*m | Nonsense | E418* | Germline | Homozygous |  |  |  |  | Myriad | Mutant |
| g*BRCA1*m | Frameshift | E23fs*17 | Germline | Homozygous |  |  |  |  | CRF | Mutant |
| g*BRCA1*m | Nonsense | Y978* | Germline | Homozygous |  |  |  |  | Both | Mutant |
| g*BRCA1*m | Frameshift | Q1756fs*74 | Germline | Homozygous |  |  |  |  | Not tested | Not tested |
| g*BRCA1*m | Frameshift | S1655fs*16 | Germline | Homozygous |  |  |  |  | CRF | Mutant |
| g*BRCA1*m  (+ VUS) | Frameshift | Q1756fs*74 | Germline | Homozygous | VUS | P1819S | Germline | Heterozygous | Both | Mutant |
| g*BRCA1*m | Frameshift | T826fs*20 | Germline | Homozygous |  |  |  |  | Both | Mutant |
| g*BRCA1*m | Frameshift | E23fs*17 | Germline | Homozygous |  |  |  |  | Both | Mutant |
| g*BRCA1*m | Frameshift | Q1756fs*74 | Germline | Homozygous |  |  |  |  | Not tested | Not tested |
| g*BRCA1*m | Frameshift | Q1756fs*74 | Germline | Homozygous |  |  |  |  | Myriad | Mutant |
| g*BRCA1*m | Frameshift | E23fs*17 | Germline | Homozygous |  |  |  |  | CRF | Mutant |
| g*BRCA1*m | Frameshift | E23fs*17 | Germline | Homozygous |  |  |  |  | CRF | Mutant |
| g*BRCA1*m | Nonsense | R1203* | Germline | Homozygous |  |  |  |  | Not tested | Not tested |
| g*BRCA1*m  (+ VUS) | Frameshift | R136fs*27 | Germline | Homozygous | VUS; VUS | M3162I; I1188M | Germline; germline | Tumor;  not in tumor | Both | Mutant |
| g*BRCA1*m | Frameshift | Q1756fs*74 | Germline | Homozygous |  |  |  |  | Both | Mutant |
| g*BRCA1*m | Nonsense | R1203* | Germline | Homozygous |  |  |  |  | Myriad | Mutant |
| g*BRCA1*m  (+ VUS) | Frameshift; VUS | E23fs*17; M658I | Germline; germline | Homozygous; not in tumor |  |  |  |  | Both | Mutant |
| g*BRCA1*m (+ VUS) | Frameshift | Q1756fs*74 | Germline | Homozygous | VUS | S1172L | Germline | Heterozygous | Both | Mutant |
| g*BRCA1*m (+ VUS) | Frameshift | K654fs*47 | Germline | Homozygous | VUS | S384F | Germline | Heterozygous | CRF | Mutant |
| g*BRCA1*m | Nonsense | Q74* | Germline | Homozygous |  |  |  |  | Myriad | Mutant |
| g*BRCA1*m | Frameshift | P1238fs*26 | Germline | Homozygous |  |  |  |  | Not tested | Not tested |
| g*BRCA1*m | Frameshift | E23fs*17 | Germline | Homozygous |  |  |  |  | Both | Mutant |
| g*BRCA1*m | Frameshift | Q1756fs*74 | Germline | Homozygous |  |  |  |  | Myriad | Non-mutant |
| g*BRCA1*m | Nonsense | E1250* | Germline | Homozygous |  |  |  |  | Both | Mutant |
| g*BRCA1*m | Frameshift | V1234fs*8 | Germline | Homozygous |  |  |  |  | Myriad | Mutant |
| g*BRCA1*m | Frameshift | S1253fs*10 | Germline | Homozygous |  |  |  |  | Both | Mutant |
| g*BRCA1*m | Frameshift | A807fs*8 | Germline | Homozygous |  |  |  |  | Both | Mutant |
| g*BRCA1*m | Frameshift | E23fs*17 | Germline | Homozygous |  |  |  |  | Both | Mutant |
| g*BRCA1*m (+ VUS) | Frameshift | S282fs*15 | Germline | Homozygous | VUS | M1149T | Germline | Heterozygous | CRF | Mutant |
| g*BRCA1*m (+ VUS) | Frameshift; VUS; VUS | Q1756fs*74; Q1227H; D411V | Germline; germline; germline | Homozygous; not in tumor; not in tumor |  |  |  |  | CRF | Mutant |
| g*BRCA1*m | Frameshift | Q1756fs*74 | Germline | Homozygous |  |  |  |  | Myriad | Mutant |
| g*BRCA1*m | Frameshift | Q1756fs*74 | Germline | Homozygous |  |  |  |  | Myriad | Non-mutant |
| g*BRCA1*m (+ VUS) | Frameshift; VUS | E765fs*1; G535R | Germline; germline | Homozygous; homozygous |  |  |  |  | Not tested | Not tested |
| g*BRCA1*m (+ VUS) | Nonsense; VUS | R1203*; L246V | Germline; germline | Homozygous; homozygous |  |  |  |  | Myriad | Mutant |
| g*BRCA1*m | Frameshift | Q1756fs*74 | Germline | Homozygous |  |  |  |  | Both | Mutant |
| g*BRCA1*m | Frameshift | D1065fs*2 | Germline | Homozygous |  |  |  |  | Myriad | Mutant |
| g*BRCA1*m | Nonsense | R1203* | Germline | Homozygous |  |  |  |  | Myriad | Mutant |
| g*BRCA1*m (+ VUS) | Frameshift; VUS | Q1756fs*74; S590G | Germline; germline | Homozygous; homozygous |  |  |  |  | Not tested | Not tested |
| g*BRCA1*m | Frameshift | E23fs*17 | Germline | Homozygous |  |  |  |  | CRF | Mutant |
| g*BRCA1*m | Frameshift | Q1756fs*74 | Germline | Homozygous |  |  |  |  | Not tested | Not tested |
| g*BRCA1*m | Nonsense | Q780* | Germline | Unknown |  |  |  |  | Both | Mutant |
| g*BRCA1*m | Frameshift | S945fs*6 | Germline | Unknown |  |  |  |  | Not tested | Not tested |
| g*BRCA1*m | Frameshift | R1835fs*10+ | Germline | Unknown |  |  |  |  | Both | Mutant |
| g*BRCA1*m | Frameshift | E23fs*17 | Germline | Unknown |  |  |  |  | CRF | Mutant |
| g*BRCA1*m | Frameshift | Q1756fs*74 | Germline | Unknown |  |  |  |  | Myriad | Mutant |
| g*BRCA1*m | Nonsense | E908* | Germline | Unknown |  |  |  |  | Both | Mutant |
| g*BRCA1*m | Frameshift | P1806fs*28 | Germline | Unknown |  |  |  |  | Myriad | Mutant |
| g*BRCA1*m | Frameshift | M1083fs*1 | Germline | Unknown |  |  |  |  | Myriad | Mutant |
| g*BRCA1*m | Nonsense | R1443* | Germline | Unknown |  |  |  |  | CRF | Mutant |
| g*BRCA1*m | Frameshift | S1428fs*6 | Germline | Unknown |  |  |  |  | Both | Mutant |
| g*BRCA1*m | Frameshift | Q1756fs*74 | Germline | Unknown |  |  |  |  | Myriad | Mutant |
| g*BRCA1*m | Frameshift | Q1756fs*74 | Germline | Unknown |  |  |  |  | Both | Mutant |
| s*BRCA1*m | Nonsense | R1203* | Somatic | Homozygous |  |  |  |  | CRF | Non-mutant |
| s*BRCA1*m | Frameshift | I600fs*7 | Somatic | Homozygous |  |  |  |  | Myriad | Non-mutant |
| s*BRCA1*m | Frameshift | S1486fs*19 | Somatic | Homozygous |  |  |  |  | Myriad | Non-mutant |
| s*BRCA1*m | Frameshift | N542fs*2 | Somatic | Homozygous |  |  |  |  | Myriad | Non-mutant |
| s*BRCA1*m | Frameshift | L1472fs*33 | Somatic | Homozygous |  |  |  |  | Not tested | Not tested |
| s*BRCA1*m | Frameshift | K50fs*19 | Somatic | Homozygous |  |  |  |  | Myriad | Non-mutant |
| s*BRCA1*m | Nonsense | E1629* | Somatic | Homozygous |  |  |  |  | Myriad | Non-mutant |
| s*BRCA1*m | Nonsense | Q1327* | Somatic | Homozygous |  |  |  |  | Myriad | Non-mutant |
| (s)*BRCA1*m | Frameshift | N1355fs*10 | NA | Unknown |  |  |  |  | Myriad | Non-mutant |
| g*BRCA1*m | Splice | Splice | Germline | Homozygous |  |  |  |  | Both | Mutant |
| s*BRCA1*m | Splice | Splice | Somatic | Homozygous |  |  |  |  | Not tested | Not tested |
| s*BRCA1*m | Splice | Splice | Somatic | Homozygous |  |  |  |  | Not tested | Not tested |
| (g)*BRCA1*m | Deletion | 0 copies |  |  |  |  |  |  | CRF | Mutant |
| g*BRCA1*m | Known-missense | R1699Q | Germline | Homozygous |  |  |  |  | Both | Mutant |
| g*BRCA1*m (+ VUS) | Known-missense | C61G | Germline | Homozygous | VUS | L1195M | Germline | Homozygous | Myriad | Mutant |
| g*BRCA1*m | Known-missense | A1708E | Germline | Homozygous |  |  |  |  | CRF | Non-mutant |
| g*BRCA1*m | Known-missense | R1699W | Germline | Homozygous |  |  |  |  | Both | Mutant |
| s*BRCA1*m | Known-missense | M1T | Somatic | Homozygous |  |  |  |  | Myriad | Non-mutant |
| g*BRCA2*m (+ VUS) | VUS | P1238L | Germline | Homozygous | Frameshift; VUS | S599fs*1; K1690N | Germline; germline | Homozygous; homozygous | CRF | Non-mutant |
| g*BRCA2*m |  |  |  |  | Frameshift | A938fs*21 | Germline | Homozygous | Myriad | Non-mutant |
| g*BRCA2*m |  |  |  |  | Nonsense | S1882* | Germline | Homozygous | Myriad | Mutant |
| g*BRCA2*m (+ VUS) |  |  |  |  | Frameshift; VUS | V1610fs*4; splice | Germline | Homozygous; not in tumor | Not tested | Not tested |
| g*BRCA2*m |  |  |  |  | Frameshift | L1466fs*2 | Germline | Homozygous | Not tested | Not tested |
| g*BRCA2*m |  |  |  |  | Frameshift | E1493fs*10 | Germline | Homozygous | Myriad | Mutant |
| g*BRCA2*m |  |  |  |  | Frameshift | S1982fs*22 | Germline | Homozygous | CRF | Mutant |
| g*BRCA2*m |  |  |  |  | Frameshift | W1692fs*3 | Germline | Homozygous | Both | Mutant |
| g*BRCA2*m |  |  |  |  | Frameshift | V1283fs*2 | Germline | Homozygous | Myriad | Mutant |
| g*BRCA2*m |  |  |  |  | Frameshift | F2254fs*6 | Germline | Homozygous | Not tested | Not tested |
| g*BRCA2*m (+ VUS) | VUS | L440S | Germline | Homozygous | Nonsense | R3128* | Germline | Homozygous | Both | Mutant |
| g*BRCA2*m |  |  |  |  | Frameshift | V1610fs*4 | Germline | Homozygous | CRF | Non-mutant |
| g*BRCA2*m |  |  |  |  | Frameshift | S1982fs*22 | Germline | Homozygous | CRF | Mutant |
| g*BRCA2*m (+ VUS) |  |  |  |  | Frameshift; VUS | V1283fs*2; I2672V | Germline; germline | Homozygous; not in tumor | Myriad | Mutant |
| g*BRCA2*m |  |  |  |  | Frameshift | Q2009fs*9 | Germline | Homozygous | CRF | Mutant |
| g*BRCA2*m |  |  |  |  | Frameshift | N1544fs*4 | Germline | Homozygous | Myriad | Mutant |
| g*BRCA2*m |  |  |  |  | Nonsense | L1118_E1119>* | Germline | Homozygous | Myriad | Mutant |
| g*BRCA2*m |  |  |  |  | Frameshift | V1804fs*2 | Germline | Homozygous | CRF | Mutant |
| g*BRCA2*m |  |  |  |  | Frameshift | Q1175fs*7 | Germline | Homozygous | Not tested | Not tested |
| g*BRCA2*m |  |  |  |  | Frameshift | S1982fs*22 | Germline | Homozygous | Both | Mutant |
| g*BRCA2*m |  |  |  |  | Frameshift | E1646fs*24 | Germline | Homozygous | Myriad | Mutant |
| g*BRCA2*m |  |  |  |  | Frameshift | L2092fs*7 | Germline | Unknown | Not tested | Not tested |
| g*BRCA2*m |  |  |  |  | Frameshift | S1982fs*22 | Germline | Unknown | CRF | Mutant |
| g*BRCA2*m |  |  |  |  | Frameshift | E1646fs*24 | Germline | Unknown | Both | Mutant |
| g*BRCA2*m |  |  |  |  | Nonsense | R2494* | Germline | Unknown | Myriad | Mutant |
| g*BRCA2*m |  |  |  |  | Frameshift | R645fs*15 | Germline | Unknown | CRF | Mutant |
| s*BRCA2*m |  |  |  |  | Frameshift | W31fs*1 | Somatic | Homozygous | CRF | Non-mutant |
| s*BRCA2*m (+ VUS) |  |  |  |  | Nonsense; VUS | K2939*; Y3098H | Somatic; germline | Homozygous; not in tumor | Not tested | Not tested |
| s*BRCA2*m |  |  |  |  | Frameshift | D1321fs*14 | Somatic | Homozygous | CRF | Non-mutant |
| s*BRCA2*m |  |  |  |  | Frameshift | D2005fs*34 | Somatic | Homozygous | CRF | Non-mutant |
| s*BRCA2*m |  |  |  |  | Frameshift | L2996fs*5 | Somatic | Heterozygous | CRF | Non-mutant |
| s*BRCA2*m |  |  |  |  | Frameshift | L2587fs*9 | Subclonal somatic | Unknown | Not tested | Not tested |
| s*BRCA2*m (+ VUS) | VUS | T826K | Germline | Homozygous | Frameshift | T2515fs*9 | Subclonal somatic | Unknown | Not tested | Not tested |
| (s)*BRCA2*m |  |  |  |  | Deletion | 0 copies |  |  | Myriad | Non-mutant |
| (g)*BRCA2*m |  |  |  |  | Deletion | 0 copies |  |  | Myriad | Mutant |
| u*BRCA2*m |  |  |  |  | Deletion | 0 copies |  |  | Not tested | Not tested |
| (g*BRCA*; FM tumor no call) | (Insertion) |  |  |  |  |  |  |  | CRF | Mutant |
| (g*BRCA*; FM tumor no call) | (Insertion) |  |  |  |  |  |  |  | Myriad | Mutant |
| (g*BRCA*; FM tumor no call) | (Deletion) |  |  |  |  |  |  |  | CRF | Mutant |
| VUS-LOH | VUS | T1700I | Germline | Homozygous |  |  |  |  | Myriad | VUS |
| VUS-LOH |  |  |  |  | VUS | N2113S | Germline | Homozygous | CRF | Non-mutant |
| VUS-LOH |  |  |  |  | VUS | D2679Y | Germline | Homozygous | Myriad | VUS |
| VUS-LOH | VUS | E649Q | Germline | Homozygous |  |  |  |  | Myriad | VUS |
| VUS-LOH |  |  |  |  | VUS | K607T | Germline | Homozygous | Not tested | Not tested |
| VUS-NA | VUS | Q804H | Germline | Unknown |  |  |  |  | Myriad | Non-mutant |
| VUS-NA | VUS | R504C | Germline | Unknown |  |  |  |  | Both | Non-mutant |
| VUS-NA  (two somatic) |  |  |  |  | VUS; VUS | E2260Q; E2275K | Somatic; somatic | Unknown; unknown | CRF | Non-mutant |
| VUS-HET |  |  |  |  | VUS | D125G | Germline | Heterozygous | Not tested | Not tested |
| VUS-NIT |  |  |  |  | VUS | N54I | Germline | Not in tumor | Both | Non-mutant |
| VUS-NIT | VUS | V1714_S1715del | Germline | Not in tumor |  |  |  |  | Myriad | Non-mutant |
| VUS-NIT | VUS | N799K | Germline | Not in tumor |  |  |  |  | Myriad | VUS |
| Non-mutant (S236R SNP) |  |  |  |  | Non-mutant VUS | S326R | Germline | Not in tumor | Myriad | Non-mutant |
| Non-mutant |  |  |  |  |  |  |  |  | Myriad | Non-mutant |
| Non-mutant |  |  |  |  |  |  |  |  | Both | Non-mutant |
| Non-mutant |  |  |  |  |  |  |  |  | Myriad | Non-mutant |
| Non-mutant |  |  |  |  |  |  |  |  | Myriad | Non-mutant |
| Non-mutant |  |  |  |  |  |  |  |  | Both | Non-mutant |
| Non-mutant |  |  |  |  |  |  |  |  | Both | Non-mutant |
| Non-mutant |  |  |  |  |  |  |  |  | Myriad | Non-mutant |
| Non-mutant |  |  |  |  |  |  |  |  | Myriad | Non-mutant |
| Non-mutant |  |  |  |  |  |  |  |  | Myriad | Non-mutant |
| Non-mutant |  |  |  |  |  |  |  |  | Myriad | Non-mutant |
| Non-mutant |  |  |  |  |  |  |  |  | Myriad | Non-mutant |
| Non-mutant |  |  |  |  |  |  |  |  | Myriad | Non-mutant |
| Non-mutant |  |  |  |  |  |  |  |  | Myriad | Non-mutant |
| Non-mutant |  |  |  |  |  |  |  |  | Myriad | Non-mutant |
| Non-mutant |  |  |  |  |  |  |  |  | Myriad | Non-mutant |
| Non-mutant |  |  |  |  |  |  |  |  | Myriad | Non-mutant |
| Non-mutant |  |  |  |  |  |  |  |  | Myriad | Non-mutant |
| Non-mutant |  |  |  |  |  |  |  |  | Myriad | Non-mutant |
| Non-mutant |  |  |  |  |  |  |  |  | Myriad | Non-mutant |
| Non-mutant |  |  |  |  |  |  |  |  | Myriad | Non-mutant |
| Non-mutant |  |  |  |  |  |  |  |  | Myriad | Non-mutant |
| Non-mutant |  |  |  |  |  |  |  |  | Myriad | Non-mutant |
| Non-mutant |  |  |  |  |  |  |  |  | Myriad | Non-mutant |
| Non-mutant |  |  |  |  |  |  |  |  | Not tested | Not tested |
| Non-mutant |  |  |  |  |  |  |  |  | Not tested | Not tested |
| Non-mutant |  |  |  |  |  |  |  |  | CRF | Non-mutant |
| Non-mutant |  |  |  |  |  |  |  |  | CRF | Non-mutant |
| Non-mutant |  |  |  |  |  |  |  |  | CRF | Non-mutant |
| Non-mutant |  |  |  |  |  |  |  |  | Both | Non-mutant |
| Non-mutant |  |  |  |  |  |  |  |  | Myriad | Non-mutant |
| Non-mutant |  |  |  |  |  |  |  |  | Myriad | Non-mutant |
| Non-mutant |  |  |  |  |  |  |  |  | Myriad | Non-mutant |
| Non-mutant |  |  |  |  |  |  |  |  | Myriad | Non-mutant |
| Non-mutant |  |  |  |  |  |  |  |  | Myriad | Non-mutant |
| Non-mutant |  |  |  |  |  |  |  |  | Myriad | Non-mutant |
| Non-mutant |  |  |  |  |  |  |  |  | Not tested | Not tested |
| Non-mutant |  |  |  |  |  |  |  |  | Not tested | Not tested |
| Non-mutant |  |  |  |  |  |  |  |  | Not tested | Not tested |
| Non-mutant |  |  |  |  |  |  |  |  | Not tested | Not tested |
| Non-mutant |  |  |  |  |  |  |  |  | Myriad | Non-mutant |
| Non-mutant |  |  |  |  |  |  |  |  | Myriad | Non-mutant |
| Non-mutant |  |  |  |  |  |  |  |  | Both | Non-mutant |
| Non-mutant |  |  |  |  |  |  |  |  | Myriad | Non-mutant |
| Non-mutant |  |  |  |  |  |  |  |  | Myriad | Non-mutant |
| Non-mutant |  |  |  |  |  |  |  |  | Not tested | Not tested |
| Non-mutant |  |  |  |  |  |  |  |  | Myriad | Non-mutant |
| Non-mutant |  |  |  |  |  |  |  |  | Not tested | Not tested |
| Non-mutant |  |  |  |  |  |  |  |  | Myriad | Non-mutant |
| Non-mutant |  |  |  |  |  |  |  |  | Both | Non-mutant |
| Non-mutant |  |  |  |  |  |  |  |  | Both | Non-mutant |
| Non-mutant |  |  |  |  |  |  |  |  | Myriad | Non-mutant |
| Non-mutant |  |  |  |  |  |  |  |  | Myriad | Non-mutant |
| Non-mutant |  |  |  |  |  |  |  |  | Both | Non-mutant |
| Non-mutant |  |  |  |  |  |  |  |  | Both | Non-mutant |
| Non-mutant |  |  |  |  |  |  |  |  | Myriad | Non-mutant |
| Non-mutant |  |  |  |  |  |  |  |  | Both | Non-mutant |
| Non-mutant |  |  |  |  |  |  |  |  | CRF | Non-mutant |
| Non-mutant |  |  |  |  |  |  |  |  | Not tested | Not tested |
| Non-mutant |  |  |  |  |  |  |  |  | Myriad | Non-mutant |
| Non-mutant |  |  |  |  |  |  |  |  | Myriad | Non-mutant |
| Non-mutant |  |  |  |  |  |  |  |  | Myriad | Non-mutant |
| Non-mutant |  |  |  |  |  |  |  |  | Myriad | Non-mutant |
| Non-mutant |  |  |  |  |  |  |  |  | Not tested | Not tested |
| Non-mutant |  |  |  |  |  |  |  |  | Myriad | Bon-mutant |
| Non-mutant |  |  |  |  |  |  |  |  | Not tested | Not tested |
| Non-mutant |  |  |  |  |  |  |  |  | Myriad | Non-mutant |
| Non-mutant |  |  |  |  |  |  |  |  | Both | Non-mutant |
| Non-mutant |  |  |  |  |  |  |  |  | Myriad | Non-mutant |
| Non-mutant |  |  |  |  |  |  |  |  | Myriad | Non-mutant |
| Non-mutant |  |  |  |  |  |  |  |  | Both | Non-mutant |
| Non-mutant |  |  |  |  |  |  |  |  | Both | Non-mutant |
| Non-mutant |  |  |  |  |  |  |  |  | Not tested | Not tested |
| Non-mutant |  |  |  |  |  |  |  |  | Myriad | Non-mutant |
| Non-mutant |  |  |  |  |  |  |  |  | Myriad | Non-mutant |
| Non-mutant |  |  |  |  |  |  |  |  | Not tested | Not tested |
| Non-mutant |  |  |  |  |  |  |  |  | Myriad | Non-mutant |
| Non-mutant |  |  |  |  |  |  |  |  | CRF | Non-mutant |
| Non-mutant |  |  |  |  |  |  |  |  | Myriad | Non-mutant |
| Non-mutant |  |  |  |  |  |  |  |  | Both | Non-mutant |
| Non-mutant |  |  |  |  |  |  |  |  | Myriad | Non-mutant |
| Non-mutant |  |  |  |  |  |  |  |  | Not tested | Not tested |
| Non-mutant |  |  |  |  |  |  |  |  | Myriad | Non-mutant |

CRF, case report form; FM, Foundation medicine; g*BRCA*m, germline *BRCA* mutation; HET, heterozygous; LOH, loss of heterozygosity; NA, not available; NIT, not in tumor; s*BRCA*m, somatic *BRCA* mutation; SNP, single nucleotide polymorphism; VUS, variant of unknown significance

**Supplemental Table 2.** Comparison of *BRCA1/2* mutations for Study 19 with three published cohorts of high-grade serous ovarian cancer patients [1-3]. Number of patients sequenced, origin of *BRCA1/2* mutation (germline, somatic, or unknown), frequencies of *BRCA1/2* mutation, and somatic mutation rates are presented. Some data for Hennessey et al. [4] are not presented here owing to testing of a subset of samples for germline and somatic status

|  | **Study 19** | **Pennington 2014 [2]** | **TCGA  2011 [1]** | **Hennessey 2010 [4]** |
| --- | --- | --- | --- | --- |
| HG-SOC patients sequenced, n | 209 | 249 | 316 | 158 |
| *BRCA1/2*m, n | 114 | 65 | 63 | 36 |
| Germline | 93 | 53 | 44^a^ | N/A |
| Somatic | 20 | 12 | 19 | N/A |
| Unknown | 1 | 0 | 0 | N/A |
| *BRCA*m, % | 55 | 26 | 20 | 23 |
| s*BRCA*m, % | 18 | 18 | 30 | 39^b^ |

^a^Subtracted three reported germline mutations (*BRCA2* K3326X is a benign polymorphism); ^b^Represents 11 somatic mutations in a subset of 28 *BRCA1/2*-mutated patients tested with no grade or histology stated. HG­SOC, high-grade serous ovarian cancer; N/A, not available; TCGA, The Cancer Genome Atlas

**References**

1 Cancer Genome Atlas Research Network. Integrated genomic analyses of ovarian carcinoma. Nature. 2011; 474:609-615.

2 Pennington KP, Walsh T, Harrell MI, Lee MK, Pennil CC, Rendi MH, Thornton A, Norquist BM, Casadei S, Nord AS, Agnew KJ, Pritchard CC, Scroggins S, et al. Germline and somatic mutations in homologous recombination genes predict platinum response and survival in ovarian, fallopian tube, and peritoneal carcinomas. Clin Cancer Res. 2014; 20:764-775.

3 Wooster R, Bignell G, Lancaster J, Swift S, Seal S, Mangion J, Collins N, Gregory S, Gumbs C, Micklem G. Identification of the breast cancer susceptibility gene BRCA2. Nature. 1995; 378:789-792.

4 Hennessy BT, Timms KM, Carey MS, Gutin A, Meyer LA, Flake DD, Abkevich V, Potter J, Pruss D, Glenn P, Li Y, Li J, Gonzalez-Angulo AM, et al. Somatic mutations in BRCA1 and BRCA2 could expand the number of patients that benefit from poly (ADP ribose) polymerase inhibitors in ovarian cancer. J Clin Oncol. 2010; 28:3570-3576.
